# Tithing programs: pathways for enhancing and improving the health status of the underprivileged

**DOI:** 10.1186/s12913-017-2753-7

**Published:** 2017-12-13

**Authors:** James K. Elrod, John L. Fortenberry

**Affiliations:** 1 Willis-Knighton Health System, 2600 Greenwood Road, Shreveport, LA 71103 USA; 2 LSU Shreveport, 1 University Place, Shreveport, LA 71115 USA

**Keywords:** Healthcare access, Community health, Medically underserved populations, Charity care

## Abstract

**Background:**

While quick and easy access to healthcare services is a reality for some, others experience significant hardships, even for receipt of the most basic health and medical care and attention. To those who effectively have been shut out of the healthcare marketplace due largely to economic deficiencies, healthcare providers engaged in the delivery of charitable services are a critical lifeline. Myriad attempts by governmental entities to remedy disparate access and shore up the delivery of healthcare services directed toward the disadvantaged have failed to close gaps, warranting pursuit of novel methods that offer potential and the hope that sufficient access might one day become a reality.

**Discussion:**

One innovative approach for enhancing and improving charitable healthcare endeavors in communities was developed by Willis-Knighton Health System. The initiative, known as the Tithing the Bottom Line program, essentially takes a portion of the health system’s earnings and directs these resources to fund pursuits that improve quality of life in the community, with the enhancement of health and wellness services for the underprivileged being a top priority. These resources magnify the efforts of establishments already endeavoring to serve those in need and create powerful synergies which positively impact the health status of disadvantaged populations. To shed light on Willis-Knighton Health System’s unique charitable initiative, this article describes its tithing program in detail, supplying operational guidance that will permit healthcare institutions to establish like programs in their communities.

**Conclusions:**

With healthcare access gaps remaining pronounced despite numerous attempts by governmental entities to realize full access, grassroots efforts remain critical to bolster health and wellness broadly in communities. Deficiencies carry dramatic consequences for both the disadvantaged and the greater communities in which they reside. The synergistic, cooperative effort realized by Willis-Knighton Health System’s tithing program offers great potential for reducing healthcare disparities, yielding healthier populations, enhanced opportunities, and better communities.

## Background

Despite significant efforts on the part of governmental entities, access to health and medical services remains elusive for many [1–4]. Disadvantaged populations, in particular, often face great hardships even for receipt of the most basic medical care and attention [5, 6]. Their limited access to healthcare services creates a vicious cycle. With preventive care services being beyond their reach, health problems are more likely to develop, and when they do appear, they often go untreated, leading to even more complex problems, up to and including death [4, 7, 8]. For individuals in such unfortunate circumstances, their primary hope for receipt of medical care and attention rests with charitable healthcare providers who are willing to offer services for little or no remuneration [9, 10]. Indeed, without such providers, the health status of these vulnerable populations would shift decidedly lower, but significant access gaps remain. This has led some entities to explore methods for shoring up access to medically underserved populations in unique and different ways [11–14].

One novel method for enhancing and improving charitable healthcare endeavors in communities was developed by Willis-Knighton Health System. The initiative, known as the Tithing the Bottom Line program, essentially takes a portion of the health system’s earnings and directs these resources to fund pursuits that improve quality of life in the community, with the enhancement of health and wellness services for the underprivileged being a top priority. This program is distinguished from traditional charity care initiatives operated by healthcare institutions in both magnitude of contribution and nature of operation [15]. Not-for-profit healthcare establishments receive a tax exemption and, in return for this privilege, they are required to provide community benefit [16, 17]. The definition of what constitutes community benefit and how much is required varies greatly, as federal regulations permit broad latitude and state regulations treat the subject with varying degrees of rigor, with most states permitting flexibility equivalent to that expressed in federal regulations [18]. Recent legislation has sought to bring additional clarity to community benefit, but the matter is anything but resolved at the moment [17, 19]. In the absence of specificity, however, a good rule of thumb is that community benefit minimally should equate with the benefit that institutions receive from their tax exempt status. Willis-Knighton Health System’s tithing program delivers charity which far exceeds the benefits afforded by its tax exempt status, something motivated by the system’s commitment to making a positive difference in its served markets. Resources supplied by the program are used to fund Willis-Knighton Health System’s own charitable care initiatives, but most uniquely, they also are directed externally toward deserving entities possessing missions which support the system’s own efforts to elevate the status and stature of community health and wellness, making for a unique method of collaboration which synergistically magnifies the potential for success [15].

To shed light on Willis-Knighton Health System’s distinctive charitable initiative, this article describes the Tithing the Bottom Line program in detail. Beyond presenting information on the program’s origins and supplying examples which demonstrate the community synergies and outreach benefits afforded, an establishment protocol is shared, permitting healthcare entities to develop like programs which create unique, grassroots partnerships that bolster the availability of health and wellness services for needy populations in communities.

### Willis-Knighton Health System and its tithing program

With origins dating to 1924, Willis-Knighton Health System is a not-for-profit, nongovernmental healthcare provider based in Shreveport, Louisiana. Delivering services across the gamut of health and wellness, the system comprehensively addresses the medical wants and needs of those living in the Ark-La-Tex region of the United States, the place where the states of Arkansas, Louisiana, and Texas converge. Through multiple hospitals, a wide array of centers of excellence, numerous general and specialty medical clinics, an all-inclusive retirement community, and more, Willis-Knighton Health System holds a position of market leadership in its region of operation.

In the late 1970s, in tandem with various growth pursuits, Willis-Knighton Health System began exploring novel methods for helping the less fortunate residing in Shreveport and vicinity. Already delivering a significant amount of charitable healthcare services to the area’s underprivileged populations, executives were desirous of seeking opportunities to maximize the impact of the system’s benevolence, permitting a better quality of life for those in need and an improved community which proactively addressed the less fortunate for the greater good of all. After significant reflection, it became apparent that reaching this particular state of the community would be impossible to do single-handedly, so explorations were channeled in search of methods which would bolster the efforts of other charitably-minded establishments in the marketplace, shoring up the state of the disadvantaged in and around Shreveport.

Many community agencies and associations which were dedicated to improving the health status of the underserved or engaged in supportive pursuits on education and social services fronts were very poorly funded, reducing their potential to make a difference. This realization led Willis-Knighton Health System’s executives to the notion of providing funding to these entities to support the advancement of their noble efforts, effectively creating partnerships for the advancement of charity. This perspective was and continues to be in keeping with contemporary guidance which suggests that partnerships and other forms of collaboration are critical for addressing the health needs of communities [3, 11, 20, 21]. It was envisioned that such funding would create powerful synergies, elevating quality of life prospects for the underserved and affording a better state of community health, wellness, and opportunity. This concept eventually was formalized by Willis-Knighton Health System, emerging as a program, established in 1979, known as Tithing the Bottom Line. The funding supplied by the tithing initiative helps to empower multiple entities, unified by a common goal to address underserved and disadvantaged populations [15].

Willis-Knighton Health System’s Tithing the Bottom Line program gets its name from the manner in which the program operates. Essentially, the system takes a portion of its annual earnings (i.e., the bottom line) and donates (i.e., tithes) it to charitable endeavors conducted both by the system and by deserving community organizations which are engaged in pursuits deemed to be supportive of the system’s mission and the greater good of the community. Executives coined the program’s name as a variant of tithing in the context of Christianity whereby parishioners voluntarily contribute or tithe a portion of their income to support their church and its mission. Although Willis-Knighton Health System is an independent, not-for-profit institution not affiliated with a religious denomination, the establishment historically has operated in a faith-based manner which has motivated its outreach efforts to serve the poverty stricken in the marketplace. Since the system operated its initiative very similarly to that of a tithe, effectively granting a portion of its income to benefit a greater cause, the tithing characterization was deemed to be fitting, yielding the unique Tithing the Bottom Line descriptor.

It should be noted that the Tithing the Bottom Line program indeed is a purely charitable endeavor. Charity care and bad debts expenses together make up uncompensated care. The two often are intermingled due to operational and reporting complexities, but they are very different. Charity care is distinguished from bad debts in that such initiatives are forwarded with no expectation of payment; bad debt expenses emerge from services rendered with payment expected but not received [22, 23]. Funding for Willis-Knighton Health System’s tithing program is separate and distinct from bad debt expenses incurred by the system. It is consciously directed toward investing in worthy causes of community benefit with no expectation of remuneration; the reward instead rests with the realization of better health and wellness in and around the marketplace.

### Selected examples

Since its establishment in 1979, Willis-Knighton Health System’s tithing program has awarded millions of dollars to support charitable healthcare, education, and humanitarian initiatives, positively impacting Shreveport and the greater region. Notably, the magnitude of contributions supplied by the system routinely exceeds that forwarded by many philanthropic entities in the marketplace. Originally tithing 10% of net income, Willis-Knighton Health System upgraded the formula to 10% of EBITDA (i.e., earnings before interest, taxes, depreciation, and amortization), permitting a greater allocation to address the burgeoning needs of the community. Several examples noted below provide useful illustrations for conveying the nature of the tithing program.

#### Willis-Knighton Health System's Project NeighborHealth

Established in 1995, Project NeighborHealth is a Willis-Knighton Health System initiative designed, developed, and operated to serve disadvantaged populations in Shreveport and the greater region. Beginning as a single clinic, Project NeighborHealth now is comprised of 11 medical establishments, placed intentionally inside or in close proximity to neighborhoods of extreme poverty, facilitating access to care and fostering medical compliance. Specifically, eight of Project NeighborHealth’s clinics are located within underprivileged neighborhoods, two are located in close proximity to challenged communities, and one unit—a mobile clinic based near a disadvantaged area—ventures into underserved communities throughout the region specifically to administer vaccinations to underprivileged children. Collectively, Project NeighborHealth serves as a critical healthcare lifeline for the less fortunate in the marketplace. Given that services are offered with no expectation of payment, Project NeighborHealth’s 11 clinics are funded exclusively by Willis-Knighton Health System’s tithing program. As this is an internal initiative of Willis-Knighton Health System, steps also are taken to maximize efficiencies. The system, for example, extensively turned to adaptive reuse in building Project NeighborHealth, permitting expansion of the network’s footprint by repurposing abandoned buildings to serve as clinics, stretching each and every charitable dollar to permit more lives to be touched [24]. In the absence of Project NeighborHealth, the vast majority of patients visiting the clinics would simply have to do without necessary medical services.

#### Northwest Louisiana Interfaith Pharmacy

The Northwest Louisiana Interfaith Pharmacy began providing vital services in 2003, following extensive planning on the part of community groups desirous of establishing a free pharmacy for individuals without the means to pay for their medications [25]. Based in Shreveport, this organization fills a critical healthcare access gap by giving the less fortunate access to medications on presentation of prescriptions, typically received from providers of charitable healthcare services in the marketplace, including Willis-Knighton Health System’s Project NeighborHealth. Since its inception, the Northwest Louisiana Interfaith Pharmacy has benefited from Willis-Knighton Health System’s generosity, with associated tithes providing great assistance in ensuring that the medication needs of disadvantaged populations in the region are addressed, shoring up community health and wellness.

#### MLK Health Center and Pharmacy

Founded in 1986, the MLK Health Center and Pharmacy, based centrally in Shreveport, provides an extensive range of healthcare and pharmacy services at no cost to uninsured or underinsured individuals residing in northwest Louisiana [26]. This private, not-for-profit charitable provider complements other institutions dedicated to aiding medically underserved populations in the marketplace, endeavoring to fill critical access gaps experienced by the less fortunate. Willis-Knighton Health System’s tithing program has long provided supportive funding for the MLK Health Center and Pharmacy, facilitating continuation of its important mission. Notably, the services of this particular entity coincide nicely with Willis-Knighton Health System’s own Project NeighborHealth initiative, creating productive synergies for improving the health and wellness opportunities available to underserved populations in the region. Its pharmacy services nicely complement those of the Northwest Louisiana Interfaith Pharmacy, further bolstering and illustrating associated synergies afforded by Willis-Knighton Health System’s tithing program.

#### Providence House

It is widely acknowledged in the health sciences literature that one’s health status is influenced by more than access to and receipt of health and medical attention. Environmental factors, such as education, shelter, nutrition, employment, and the like also play significant roles in health and wellness [27–29]. As such, Willis-Knighton Health System’s tithing program supports efforts that extend beyond the provision of healthcare services to the disadvantaged. The program’s long-running support directed toward Providence House, a Shreveport-based charity, nicely illustrates this. Established in 1989, Providence House endeavors to assist homeless families by providing housing, education, and related resources to help them escape poverty and homelessness [30]. Even with adequate access to healthcare services, the burden of homelessness clearly creates myriad challenges in attending to personal health matters, thus warranting Willis-Knighton Health System’s investment in the worthy mission of Providence House, providing essential support for reducing the prevalence of homelessness in and around the marketplace, ultimately facilitating elevated community health and wellness.

#### Food Bank of Northwest Louisiana

The Food Bank of Northwest Louisiana supplies another example of an organization that is not involved in the provision of healthcare services, yet plays a critical role in health and wellness. Since opening in 1997, the establishment has worked to fight hunger in northwest Louisiana, sorting, warehousing, and distributing food to a wealth of partner organizations, with all parties dedicated to ensuring that the needy have proper nourishment [31]. Hunger has vast implications for health and well-being, prompting Willis-Knighton Health System to actively support the Food Bank of Northwest Louisiana through its tithing program. Synergies here are very interesting in that by helping to bolster proper nutrition among the indigent, health and wellness is improved. This provides an enhanced quality of life for those receiving food, but it also reduces hospital admissions resulting from malnourishment and related conditions, which ultimately would consume significantly more charitable healthcare dollars than that required by the upfront investment needed to ensure that the disadvantaged receive appropriate nourishment. Investments here quite obviously benefit the poor, but they also benefit Willis-Knighton Health System by reducing the burdens on its healthcare infrastructure.

#### Regional health and medical educational institutions

Willis-Knighton Health System’s tithing program contributes significant resources to bolster health and medical education. Tithes have been forwarded to support a wealth of offerings provided by educational institutions in the region, notably including programs in medicine, health administration, and nursing at LSU Medical School, LSU Shreveport, and Northwestern State University of Louisiana, respectively. By supplying funds to establish or bolster healthcare-related academic and technical programs through professorships, salary support for faculty recruitment and retention, scholarships, and the like, the supply of individuals capable of serving proficiently in health and medical roles is increased. While the funding provided is not explicitly associated with elevating the health status of the indigent, Willis-Knighton Health System has observed many associated, positive impacts, as these institutions supply highly-qualified graduates, with many deciding to pursue opportunities in area establishments dedicated to indigent care. Many of the educational programs receiving support from Willis-Knighton Health System’s tithing initiative would be scaled back or eliminated entirely without the associated funding, diminishing the labor force and negatively impacting health access opportunities available to area residents, including disadvantaged populations. Unquestionably, the state of indigent care in the region would be reduced without Willis-Knighton Health System’s investment in regional health and medical educational institutions.

### Program establishment

**Fig. 1:**
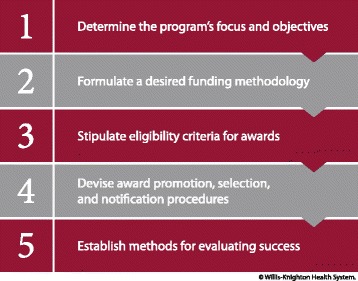
Steps for establishing a tithing program

With Willis-Knighton Health System’s Tithing the Bottom Line program approaching nearly four decades of continuous operation, executives have observed many successes which have dramatically improved quality of life in the community, with health and wellness services for the underprivileged being enhanced considerably. The synergies anticipated at the onset of the tithing program indeed were realized and continue to thrive in present day. As with most any grassroots effort, greater participation and involvement within and across communities is essential for magnifying associated impact. Missions to shore up access for the disadvantaged are no exception. In the hope that tithing programs might one day become commonplace, it is worthy to recap the steps that Willis-Knighton Health System followed for program establishment as this might help other healthcare institutions to develop similar initiatives which together will aid in resolving long-standing care access problems experienced by the underprivileged. Associated steps of establishment, outlined in Fig. 1, coupled with Willis-Knighton Health System’s experiences and insights, are explained as follows.

#### Step 1: determine the program's focus and objectives

At the onset of establishing a tithing program, it is essential to determine the initiative’s focus and associated objectives. Willis-Knighton Health System’s interest revolved around improving quality of life in the community, with indigent care being a top priority. This direction emerged from experiences and observations within the Shreveport marketplace which indicated gaps, especially regarding healthcare access opportunities available to the underprivileged. Executives studied the gaps more extensively, compelling them to develop a program which focused not only on healthcare service provision, but also on environmental matters which impact health and wellness (e.g., housing, education, employment), broadening the types of programs warranting investment. As areas of interest and associated capabilities vary from institution to institution and marketplace needs differ from community to community, it is imperative for entities desirous of establishing tithing programs to carefully evaluate their capabilities and desires in the context of community needs, selecting foci and objectives, accordingly.

#### Step 2: formulate a desired funding methodology

With the focus and objectives of the tithing program determined, attention is then directed toward the funding methodology. Willis-Knighton Health System desired to establish a program that would be enduring, yet not obligate the institution if any unforeseen circumstance was encountered in future years, something that all healthcare entities must consider, given the nature of highly-competitive and ever-changing environments. This led the system to tie the tithing program’s annual funding amount to a percent of earnings. Fortunately, Willis-Knighton Health System’s success has permitted tithes each year since the program’s inception in 1979. As noted earlier, magnitude of the system’s tithe has actually increased, moving from the original tithing formula of 10% of net income to a more robust formula of 10% of EBITDA, a step viewed as essential, given the chronic needs for support evident in the marketplace.

It should be noted that value exists in most any contribution. If an institution does not feel as though it has the ability to commit to a tithe, which implies that contributions will be ongoing subject to acceptable financial performance, it certainly can modify its approach, accordingly. Perhaps a one-time contribution of a particular amount with the option of continuing it in future years is doable or perhaps the institution could pledge nonmonetary support, such as supplying physicians to help cover weekend hours at an indigent medical clinic in the community. Regardless of approach, whether an ongoing tithe, a one-time contribution, a nonmonetary gift, or something else of value, benefits will be afforded to recipient organizations, helping to advance their missions and the mission of the associated funding entity.

#### Step 3: stipulate eligibility criteria for awards

In Willis-Knighton Health System’s experience, regardless of amount tithed, community needs always will exceed available resources, making it imperative to carefully define eligibility criteria, something which will help direct efforts and ensure that funds are being routed to candidates most likely to use the resources well. In keeping with Willis-Knighton Health System’s not-for-profit status, it was decided that entities eligible for funding consideration must be public agencies, schools, or charitable, not-for-profit organizations operating in one or more communities where a Willis-Knighton Health System facility is located, with preferences being given to health, education, and humanitarian service initiatives. This particular approach offered sufficient flexibility to direct funds as community needs evolve and also complied with requirements associated with disbursing funds as a not-for-profit, charitable enterprise. Such determinations are institution dependent and must be decided by those most familiar with the given organization and its community. The best advice for establishments first structuring their associated tithing programs is to consider pathways, stipulate eligibility criteria believed to be well suited for given programs, and as experience is gained, modify the criteria as needed to ensure that the most deserving candidates are receiving available funds.

#### Step 4: devise award promotion, selection, and notification procedures

With eligibility criteria stipulated, award promotion, selection, and notification procedures must be devised. Promotion can be active, utilizing advertisements and the like, or passive, relying on subtle conveyances and word-of-mouth communications in the community. Willis-Knighton Health System opted for a passive approach. Today, having operated its tithing program for decades, the community is very much aware of Willis-Knighton Health System’s charitable endeavor, with references noted on its website and in corporate communications providing sufficient publicity. As might be expected, the program’s lengthy history of operation has resulted in true and enduring partnerships with various establishments, with many recipients receiving funding year after year.

Given infinite community wants and needs, coupled with finite charitable resources, selection criteria must be in place to guide award distribution. Step 3 narrowed down potential award candidates by specifying eligibility criteria, but further mechanisms must be in place for deciding which applicants, among those eligible, will be selected for awards. To address these and related matters, Willis-Knighton Health System organized a committee specifically charged with responsibilities to meet annually to review applications, select award recipients, and announce decisions. Operating under the direction of the Office of the President and CEO and consisting of a range of key Willis-Knighton Health System executives, the WK Contribution Committee ensures that the given year’s gifts are directed toward causes that are deemed to be of greatest importance across served markets. To ensure that the committee possesses appropriate information for making decisions, applicants are required to submit a formal application describing the organization’s mission, the funding amount desired, the purpose for which the funds will be used, the community benefit afforded by the given pursuit, and other relevant details. It then is a matter of convening meetings as needed to review applications and make awards decisions, which then are conveyed via letter to each applicant. Wants and needs in virtually any community run exceptionally wide and deep, so great care must be taken to sift through applications in order to identify the best prospects. Willis-Knighton Health System has verified over many years the value of assembling a committee for related duties and advises the same for any institution considering the establishment of a tithing program.

#### Step 5: establish methods for evaluating success

As with any program, methods should be established for evaluating the success of tithing initiatives. Willis-Knighton Health System’s evaluation procedures are minimalistic, due to the significant efforts of the WK Contribution Committee to vet applicants. Funds are awarded only if committee members feel confident about the applicant and the intended use of the requested gift. While Willis-Knighton Health System has no requirement for award recipients to provide evidence of performance, committee members, through their high involvement in the marketplace, do have opportunities to observe the efforts of recipient entities and these perspectives are factored into consideration if award recipients seek future funding. Despite the absence of an evidence of performance requirement, however, the vast majority of award recipients do forward details of use, the parties benefiting, and their associated appreciation. Evidence requirements are subject to the desires of the institutions forwarding charitable gifts. Regardless of approach, whether rigorous or subtle, entities desirous of beginning a tithing program should ensure that recipients indeed are following through on their promises, providing assurances that precious charitable dollars are not wasted.

## Conclusions

Willis-Knighton Health System’s Tithing the Bottom Line program represents a unique approach for reducing healthcare access gaps. It funds the system’s own charitable efforts, but importantly extends community charity by tithing a portion of earnings to deserving establishments endeavoring to improve quality of life in the marketplace. In essence, the program helps others to help others, with associated synergies effectively creating broad partnerships of like-minded entities dedicated to improving community health and wellness. Tithes forwarded by the system have extensively afforded improved and enhanced healthcare access opportunities for the underprivileged in Shreveport and the greater Ark-La-Tex region of the United States.

As Willis-Knighton Health System discovered many years ago and continues to observe in present day, charitable care initiatives remain essential for achieving the best community health outcomes. With prominent gaps remaining even in light of recent governmental efforts to improve healthcare access, including mandatory health insurance initiatives, it is now more important than ever for communities to come together to address shortcomings. Willis-Knighton Health System’s tithing program offers an example of a community-based initiative which is delivering tangible results. Opportunities abound for like programs to be offered by healthcare institutions of all types and sizes and it is hoped that the overview and guidance supplied in this article will compel their development and operation across communities. Such grassroots efforts individually and especially collectively have great potential for reducing healthcare disparities, yielding healthier populations, enhanced opportunities, and better communities.
